# Supplementary material for: A Multi-omic Integrative Scheme Characterizes Tissues of Action at Loci Associated with Type 2 Diabetes
Source: Am J Hum Genet. 2020 Nov 12;107(6):1011–28. doi: 10.1016/j.ajhg.2020.10.009 (PMC7820628; doi:10.1016/j.ajhg.2020.10.009)
Supplement: Document S1. Figures S1–S10 [file mmc1.pdf]

The American Journal of Human Genetics, Volume *107*

Supplemental Data

A Multi-omic Integrative Scheme Characterizes Tissues of Action at Loci Associated with Type 2 Diabetes

Jason M. Torres, Moustafa Abdalla, Anthony Payne, Juan Fernandez-Tajes, Matthias Thurner, Vibe Nylander, Anna L. Gloyn, Anubha Mahajan, and Mark I. McCarthy

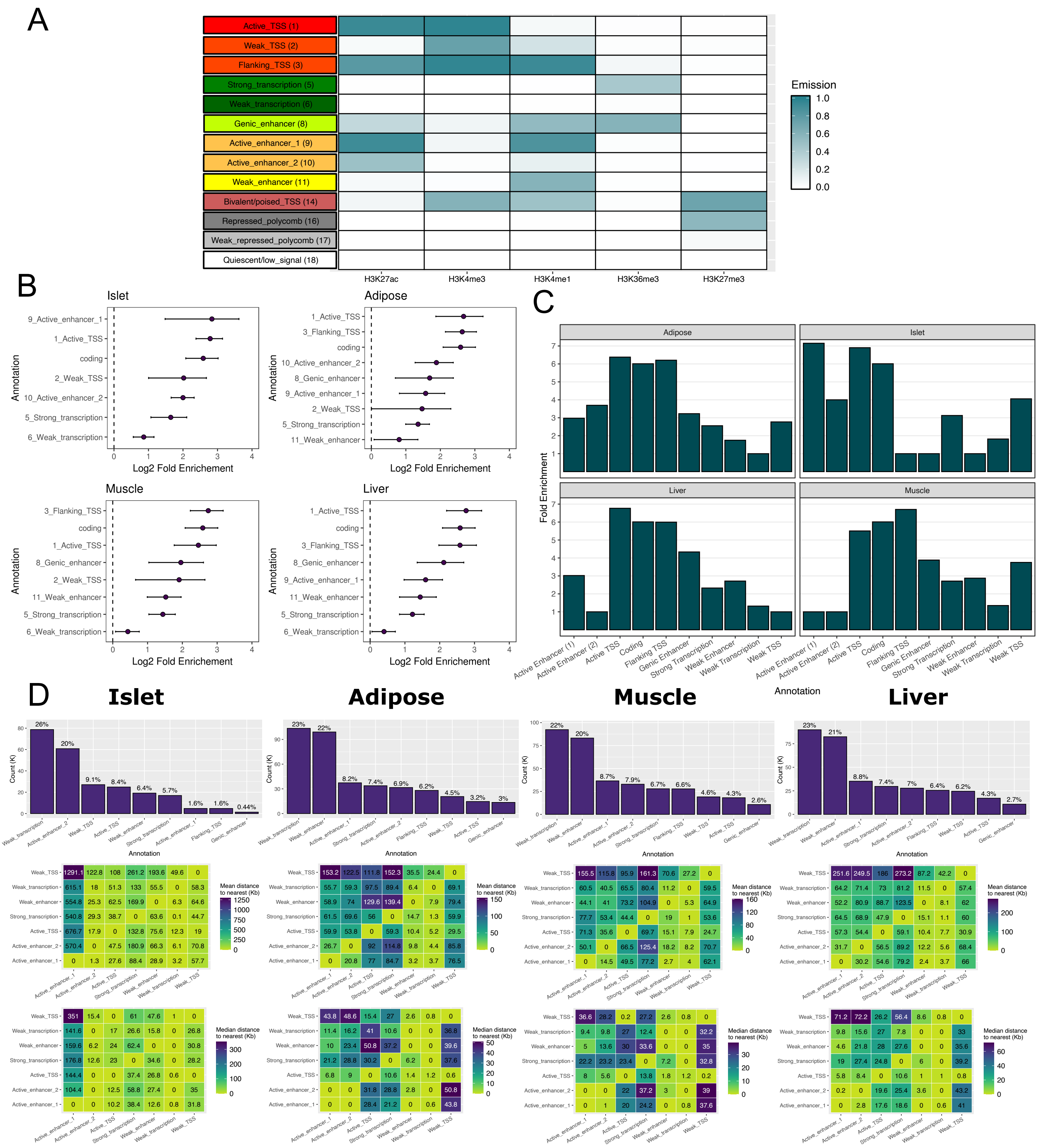

Figure S1. Epigenomic annotations are enriched for variants associated with type 2 diabetes. A) ChromHMM emission values from Varshney et al. 2017. are indicated for each chromatin state annotation. **B)** Chromatin state annotations were assessed for genome-wide enrichment of T2D-associated SNPs using the program *fgwas*. Log$_2$ fold enrichment values and 95% confidence bounds are shown for each chromatin state annotation meeting statistical significance **C)** The mean fold enrichment values for each tissue annotation were used as weights for scaling the relative contribution of each annotation vector in calculating TOA scores. **D)** The count distribution of epigenomic annotations is shown for each tissue along with heat plots showing the pairwise mean and median distances to the nearest annotation in kilobases (Kb).

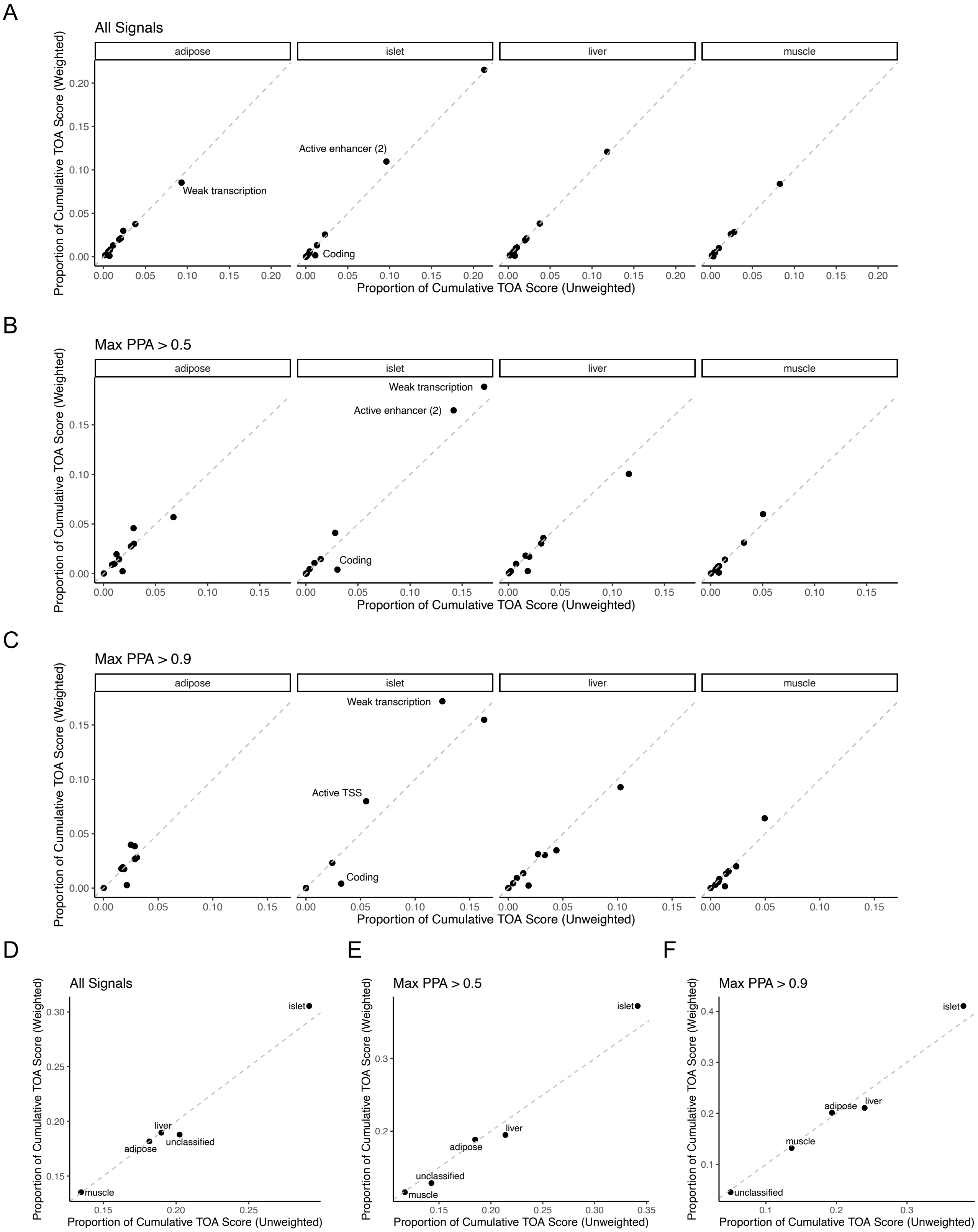

Figure S2. Comparison of GWAS effect size weighted and unweighted TOA scores. TOA scores weighted by |β|/SE, corresponding to the absolute value of the GWAS effect size divided by standard error of the sentinel SNP corresponding to each signal, are compared with the unweighted TOA scores. The proportion of cumulative score for unweighted (x-axis) and weighted (y-axis) TOA scores are shown for each annotation group in each tissue and is shown for **(A)** all signals, **(B)** all signals with maximum credible set PPA > 0.5, and **(C)** all signals with maximum credible set PPA > 0.9. The three annotation groups with the greatest differences between weighted and unweighted TOA scores, across all tissues, are indicated. The proportions of cumulative TOA scores combined across annotation groups for each tissue are shown for **(D)** all signals, **(E)** all signals with maximum credible set PPA > 0.5, and **(F)** all signals with maximum credible set PPA > 0.9.

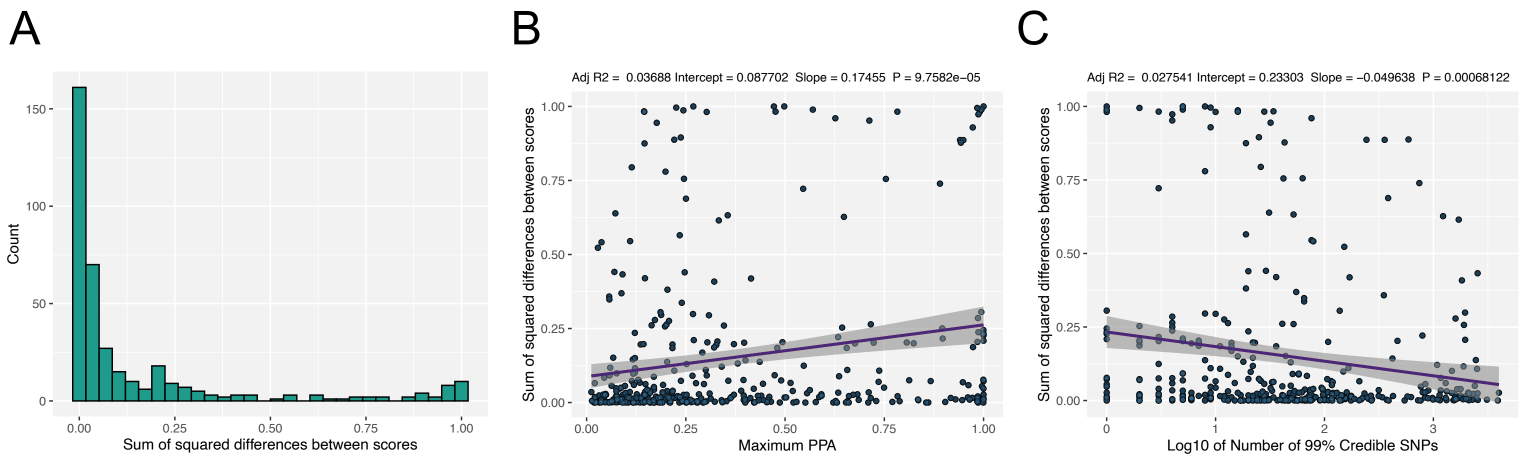

Figure S3. The relationship between credible set resolution and tissue specificity. A) Histogram of sum of squared differences (SSD) between tissue-of-action scores for islet, subcutaneous adipose, skeletal muscle, and liver across all 380 fine-mapped signals. **B)** Scatter plot of maximum 99% credible set PPA (x-axis) and SSD values (y-axis) is shown for all signals with regression line. **C)** Scatter plot of base 10 logarithm of number of 99% credible SNPs (x-axis) and SSD values (y-axis) is shown for all signals with regression line.

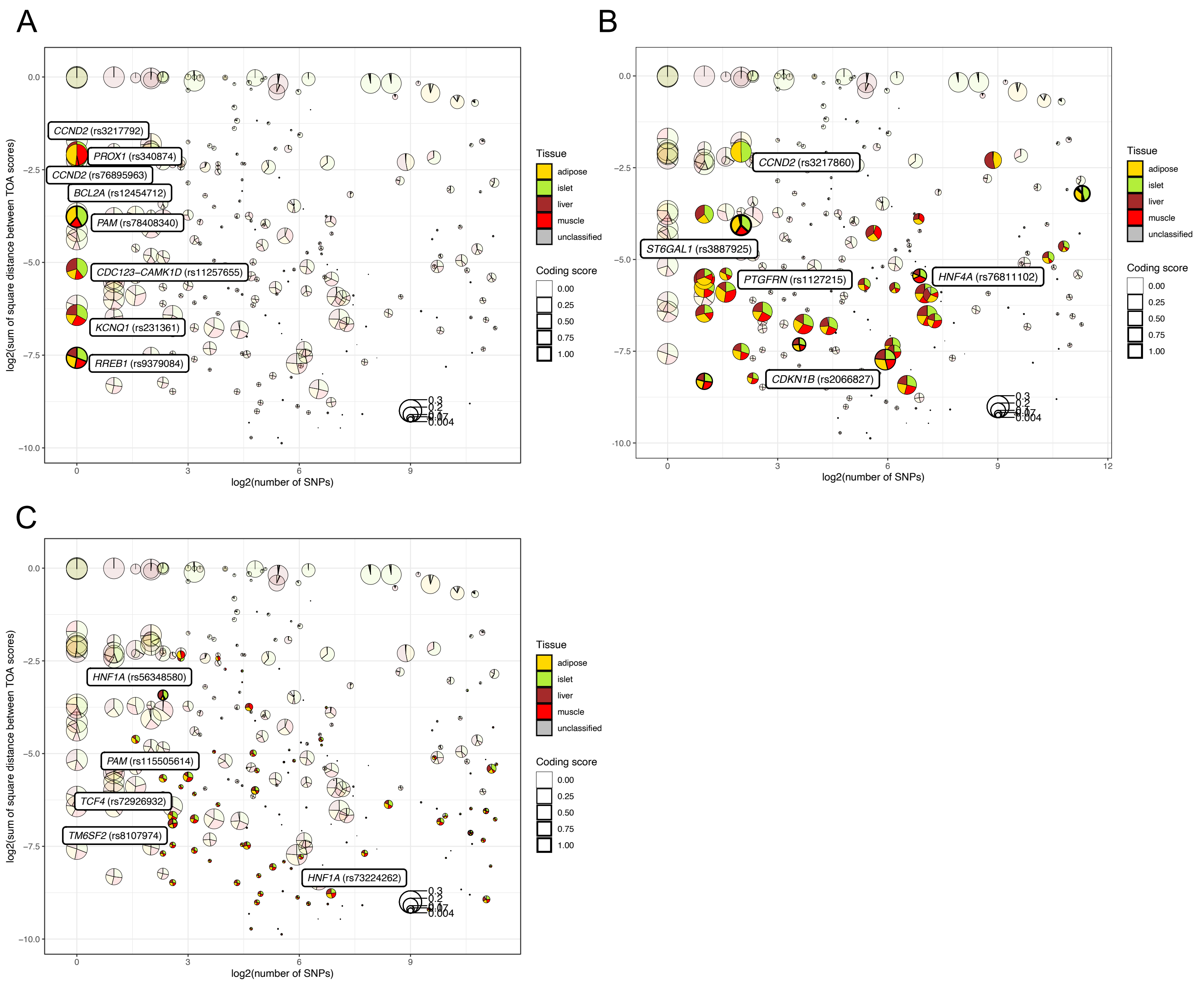

Figure S4. Fine-mapping resolution and TOA score diversity at shared signals. A) Signals that have been fine-mapped to a single credible variant. **B)** Signals with more than one credible variant but maximum PPA > 0.5. **C)** Signals with more than one credible variant and maximum PPA ≤ 0.5. Log_2 of the number of credible SNPs for each fine-mapped signal is shown on the x-axis and the log_2 value of the sum of square differences between TOA scores for each signal is shown on the y-axis (i.e. higher values on the y-axis correspond to greater tissue “specificity”). The profile of TOA scores are indicated within pie charts where the diameter of each circle corresponds to the maximum PPA for the credible set. The line thickness for each circle indicates a coding score for each credible set (i.e. the proportion of cumulative PPA attributable to coding variants).

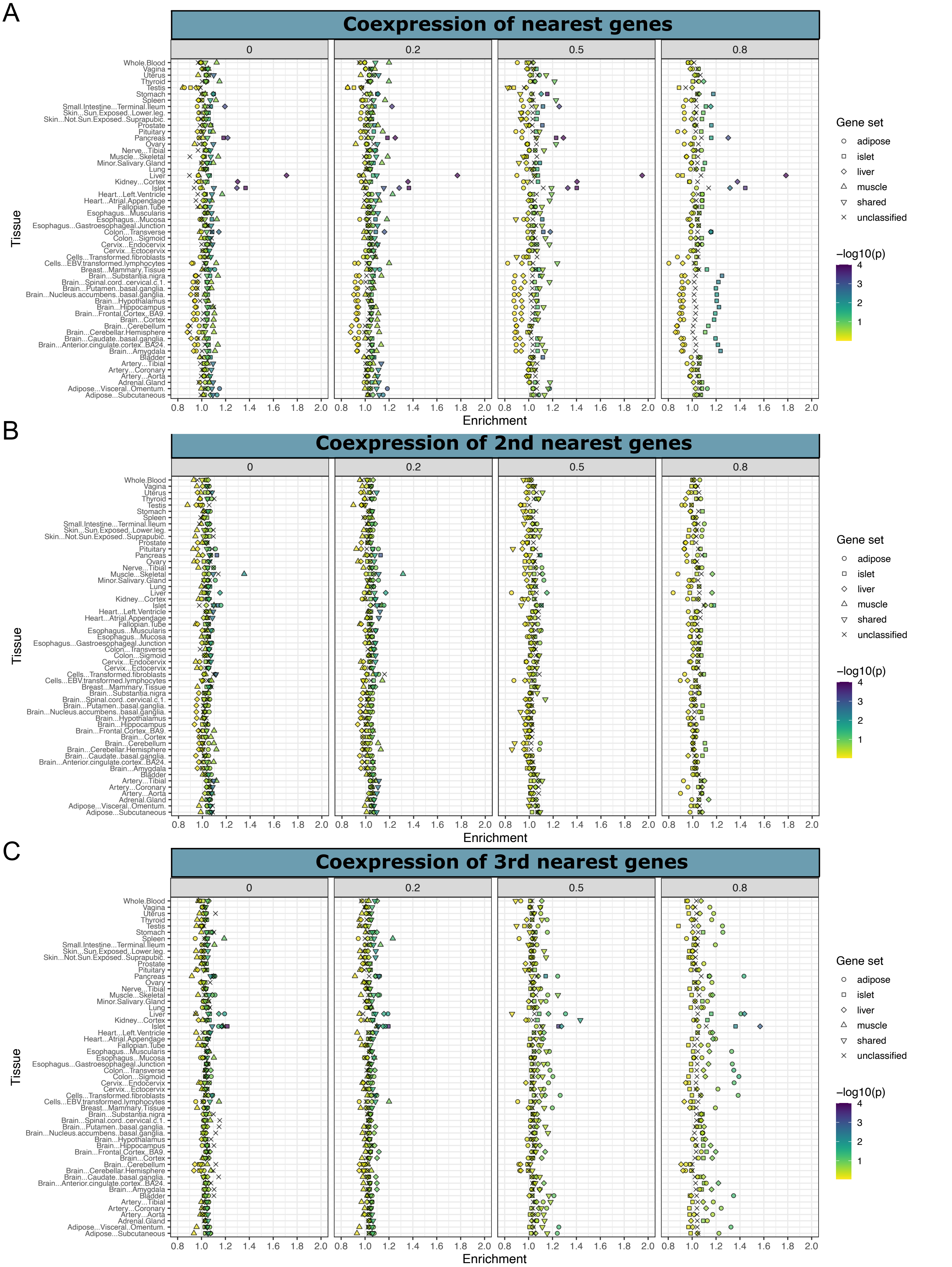

Figure S5. Expression similarity of proximal genes to tissue-assigned signals. A) Significance in similarity of expression levels of nearest genes annotated to sets of tissue-assigned signals across TOA score stringency thresholds. Corresponding values for sets of second nearest and third nearest genes are shown in **B)** and **C)**, respectively. Results are shown for human islet and 53 tissues from the Genotype-Tissue Expression Project (GTEx). Shape indicates the tissue to which the set of signals were assigned.

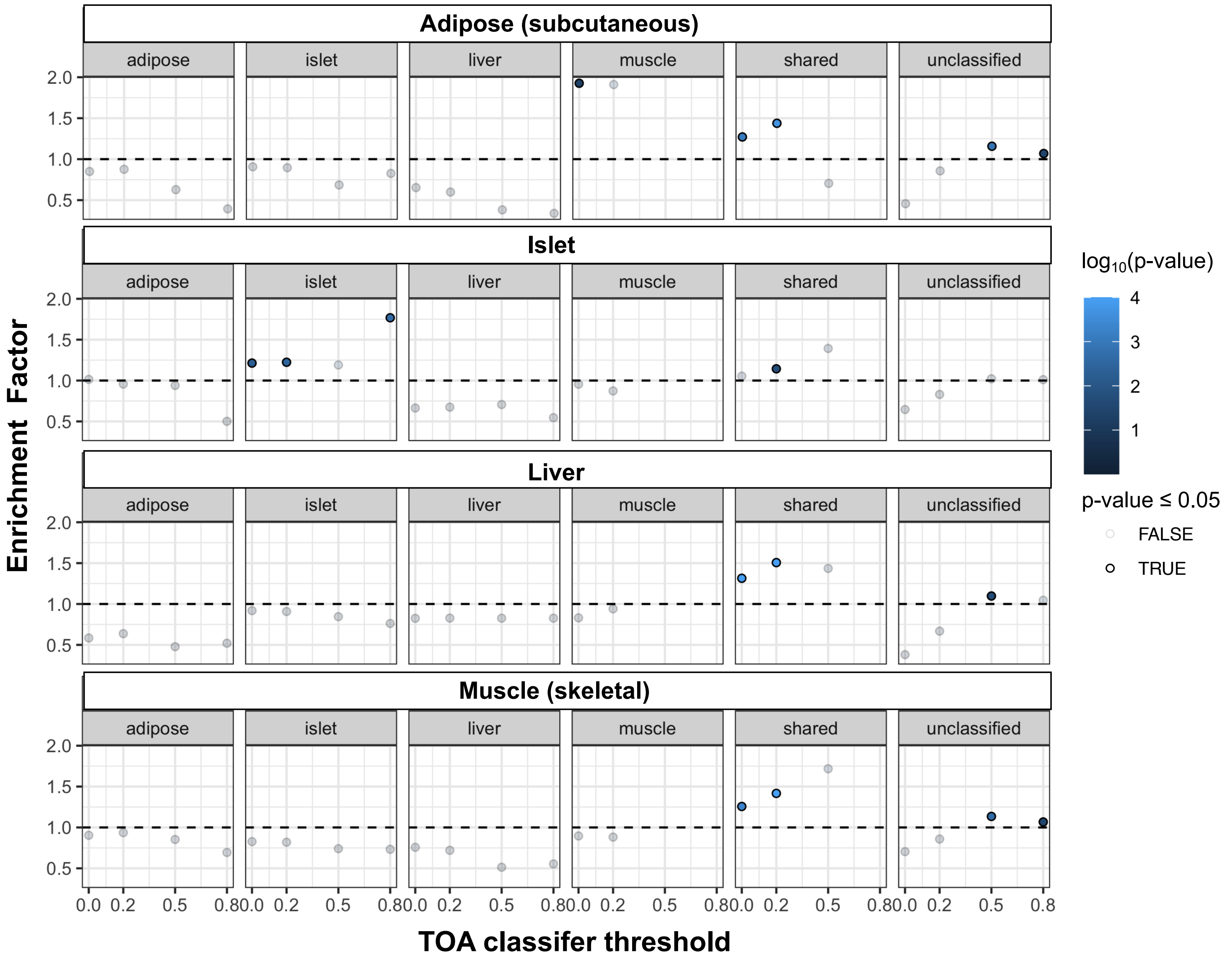

Figure S6. Co-expression of proximal genes to tissue-assigned signals. Enrichment factors from a Spearman correlation-based test of gene co-expression are shown for each set of proximal genes annotated to tissue-assigned signals, at each evaluated stringency threshold. Gene co-expression was evaluated in subcutaneous adipose, islet, liver, and skeletal muscle tissue.

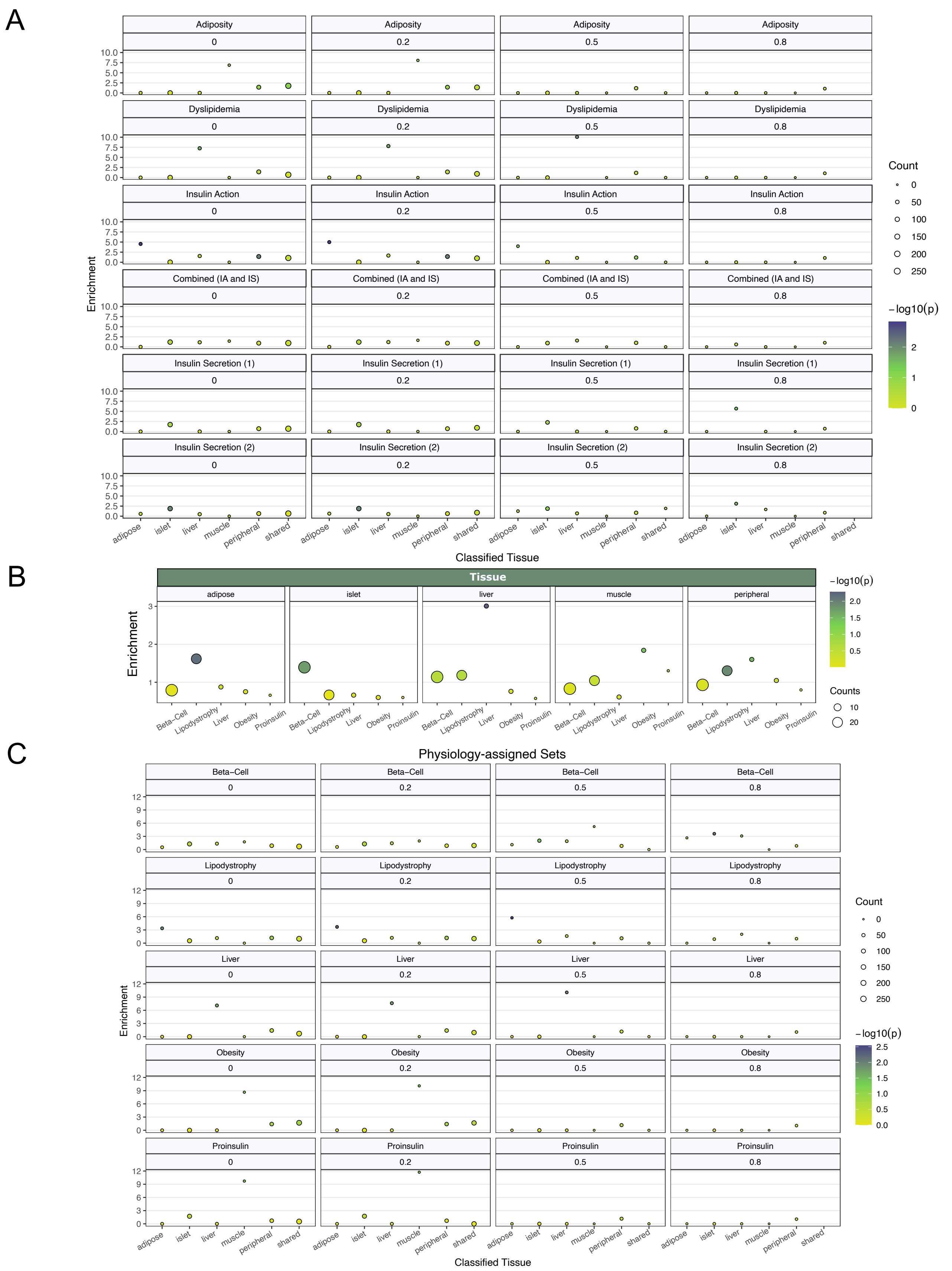

Figure S7. Comparison of sets of epigenomic- and physiology-assigned signals. A) Enrichment of 82 physiology-assigned signals, from six physiology groups, among sets of epigenomic-assigned signals based on TOA scores. Size corresponds to the number of physiology signals among each set of tissue-assigned signals. **B)** Comparison of TOA scores - as enrichments of mean observed value over a sampled mean - among sets of physiology-assigned signals from Udler et al. 2018. The size corresponds to the number assigned signals in each physiology group. **C)** Enrichment of 62 physiology-assigned signals from Udler et al. 2018., from five physiology groups, among sets of epigenomic-assigned signals based on TOA scores. Size corresponds to the number of physiology signals among each set of tissue-assigned signals.

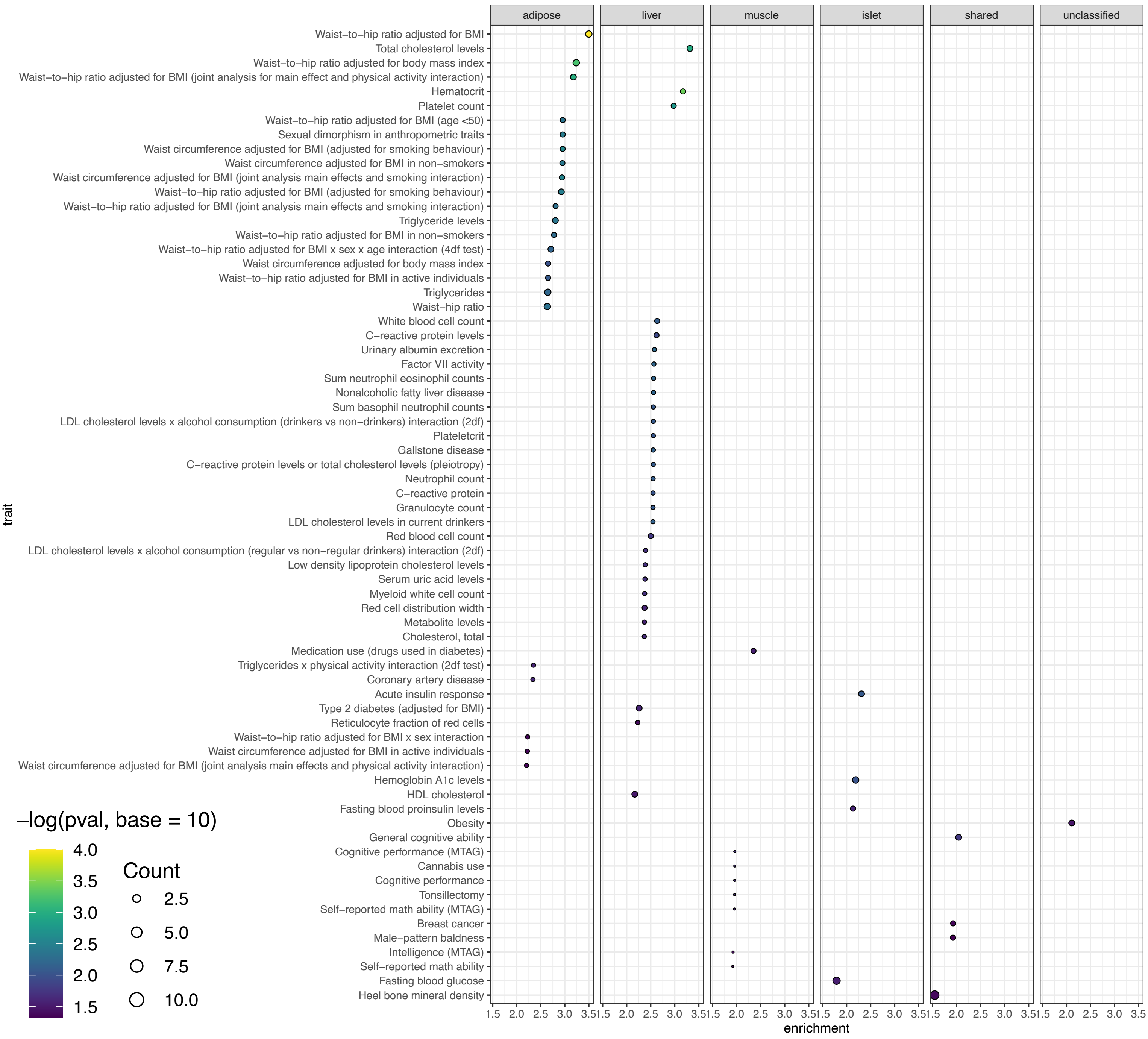

Figure S8. Enrichment of trait-associated SNPs. The results from an enrichment test are shown each trait from the NHGRI-EBI GWAS catalogue where trait associated SNPs were enriched among sets of tissue-assigned signals at a p-value ≤ 0.05. All results correspond to signals classified at the 0.2 TOA score threshold. Color corresponds to the negative $\log_{10}$ p-value and the text size corresponds to the number of SNPs that overlap lead SNPs (through identity or LD proxy at $r^2 \geq 0.8$) for each tissue-assigned signal.

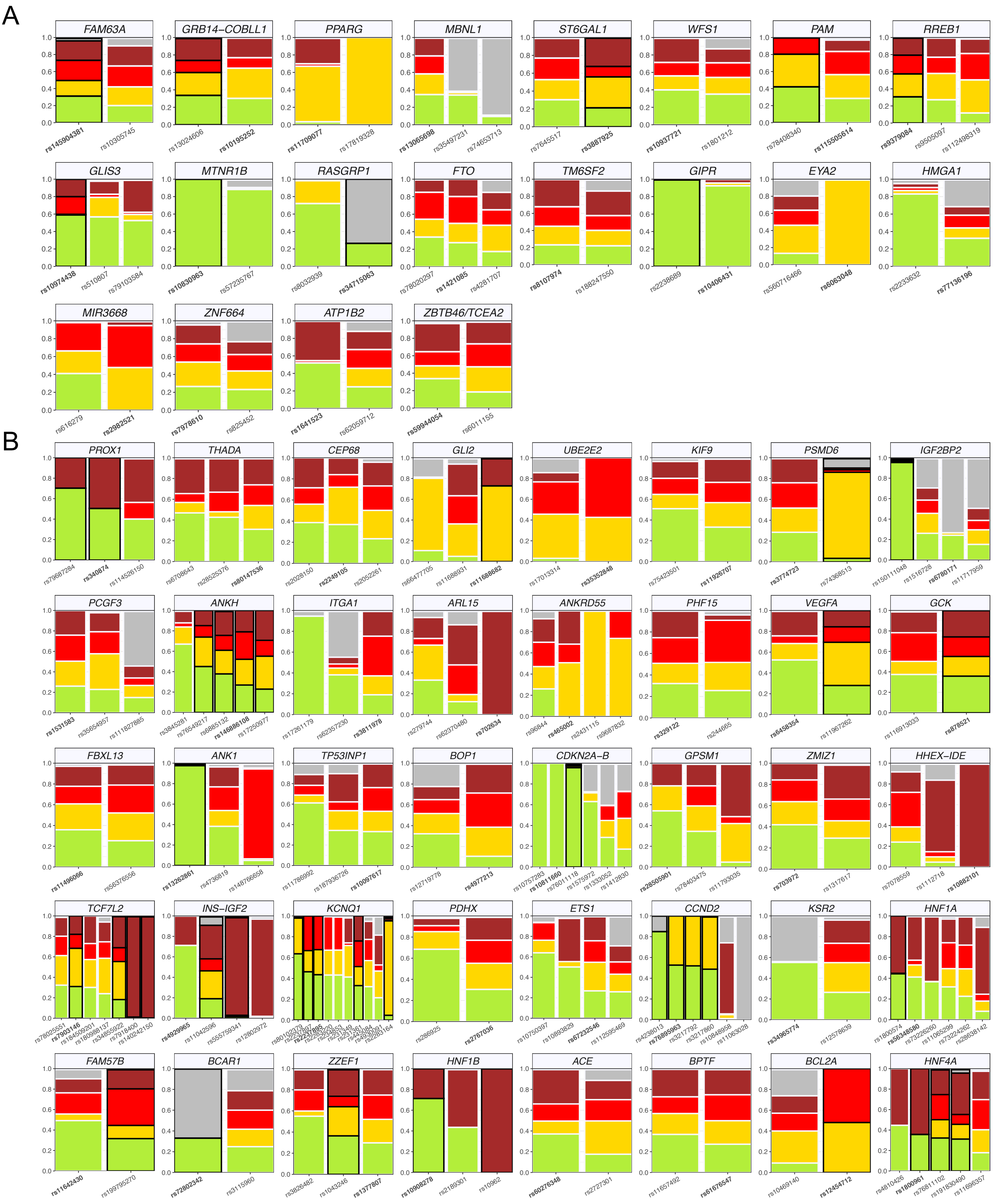

Figure S9. Multiple tissues implicated by epigenomic scores at heterogenous loci. A) Profile of TOA scores for the 20 loci with all signals receiving identical tissue assignments at the 0.2 stringency threshold **B)** Profile of TOA scores for the 40 loci with all signals receiving distinct tissue assignments at the 0.2 threshold.

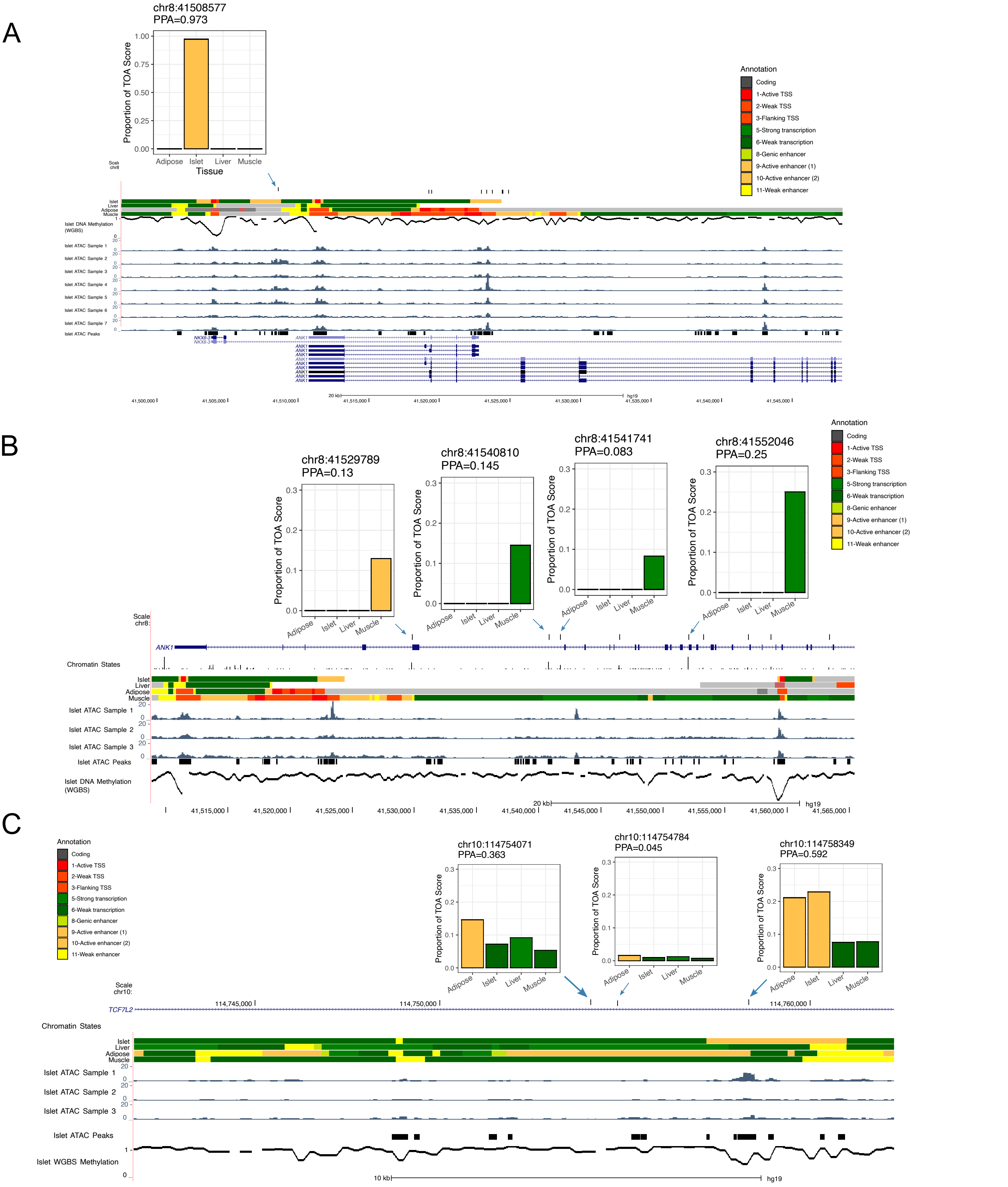

Figure S10. Epigenomic profiles of fine-mapped signals at T2D-associated loci. A) Islet-assigned and primary signal (rs13262861) at the *ANK1* locus. **B)** Muscle-assigned signal at the *ANK1* locus (rs148766658). **C)** Islet-assigned and primary signal (rs7903146) at the *TCF7L2* locus. For each credible SNP, the PPA value attributable to each tissue annotation is shown along with its position on chromosome 10 (genome build hg19). Chromatin state maps for islet, adipose, muscle, and liver tissue from Varshney et al. 2017. are shown along with ATAC-seq tracks for representative islet samples, called ATAC-seq peaks from a set of islet ATAC samples (n=17), and DNA methylation (whole genome bisulfite sequencing) in human islets from Thurner et al. 2018.
